# Characterization of the complete mitochondrial genome of brown barracuda, *Sphyraena pinguis* (Perciformes: Sphyraenidae)

**DOI:** 10.1080/23802359.2020.1797588

**Published:** 2020-07-28

**Authors:** Nazia Tabassum, Wongyu Park, Hae-Ja Baek, Jae-Young Je, Hyun-Woo Kim

**Affiliations:** a Industrial Convergence Bionix Engineering, Pukyong National University, Busan, Republic of Korea; b Department of Marine Biology, Pukyong National University, Busan, Republic of Korea; c Department of Marine-Bio Convergence Science, Pukyong National University, Busan, Republic of Korea

**Keywords:** Barracuda, Miseq, mitogenome, Korea, *Sphyraena pinguis*

## Abstract

The entire mitochondrial genome sequence of *Sphyraena pinguis* collected from Korean water was determined by the Next Generation Sequencing (NGS) technology. Its total length was 16,620 bps in length, which possessed the canonical 37 genes in the eukaryotes. Unusual start codon was exclusively found in COX1(GTG), while incomplete stop codons (TA–/T—) were identified in ATP6, COX2, ND3, ND4, and Cyt b. A phylogenetic analysis with currently identified full mitogenomes in Perciformes, *S. pinguis* was most closely related to S. *barracuda* (76.87%) and S. *jello* (76.84%). This mitogenome sequence would explain the evolution of genus *Sphyraena*.

According to FishBase (www.fishbase.org), *Sphyraena pinguis* (Günther 1874) is one of 28 species recognized in the genus Sphyraena. This species can be distinguished from the other relatives by two rakers in the first gill arch and the extended end tip in its pectoral fin (Karna et al. 2018). Although it is widely distributed in the Pacific Ocean along with Australia, Japan, and China, the genetic information of *S. pinguis* in Korean waters is still limited. We here report the first complete mitochondrial genome of *S. pinguis* determined by high-throughput sequencing (HTS) analysis.

Specimen was collected from the Southern coastal water of Korean peninsula (34°21′25.7″N 128°22′37.9″E) during a research survey funded by the Ministry of Oceans and Fisheries. Its species was identified both by the sequence identity in COI region (99.84%, GenBank number: MK560560) and its morphological characters. The specimen with its DNA is stored at the Marine Biodiversity Institute of Korea (MABIK GR00004104). Mitochondrial DNA was extracted using a commercial kit (Abcam, UK), which was furthr sheared by Covaris M220 Focused-Ultrasonicator (Covaris Inc., San Diego, CA). A library for the MiSeq platform (Illumina, San Diego, CA) was constructed with TruSeq® RNA library kit (Illumina, San Diego, CA). Geneious^®^ 11.0.2 software (Kearse et al. 2012) was employed for assembling raw reads constructing the circular mitogenome sequence of *S. pinguis*. Secondary structures of 22 tRNAs were obtained by tRNAScan-SE software (Lowe and Chan 2016). A region with low fidelity from 2500 to 5500 bp was reconfirmed by Sanger’s sequencing method. Phylogenetic tree of *S. pinguis* with its relatives was analyzed based on the Minimum evolution algorithm with 1000 bootstrap replicates (Kumar et al. 2016).

The whole mitogenome sequence of *S. pinguis* (GenBank Number: MN967008) was 16,620 bps in length, which included 13 protein-coding genes (PCG), 22 tRNAs, two ribosomal RNAs, and a control region (D-Loop). A Slightly higher overall A + T contents (52.39%) were observed compared with G + C contents (47.61%). Twenty-eight genes were located on the heavy (H) strand, while nine were on the light (L) one. Unusual start codon was exclusively found in COX1 (GTG), while incomplete stop codons (TA–/T––) were identified in ATP6, COX2, ND3, ND4, and CytB. The base composition of 832 bp long control region was 29.7% (A), 29.4% (T), 16.7% (G), and 24.2% (C), respectively. Among the compared 22 tRNAs, only the tRNA^Ser-GCT^ were predicted not to form a typical clover-leaf structure. The phylogenetic tree showed that *S. barracuda* (AP006828) and *S. jello* (KT445895) were among the most closely related to *S. pinguis* with relatively low identity (∼76.8%) (Figure 1). Especially, 16S rRNA gene to tRNA^Trp^ region gene (from 2785 bp to 5223 bp) showed low sequence identity to its relatives but the predicted gene organization of the three species were identical. The complete mitochondrial genome sequence of *S. pinguis* would be useful to understand the evolutional relationship in the genus.

**Figure 1.**
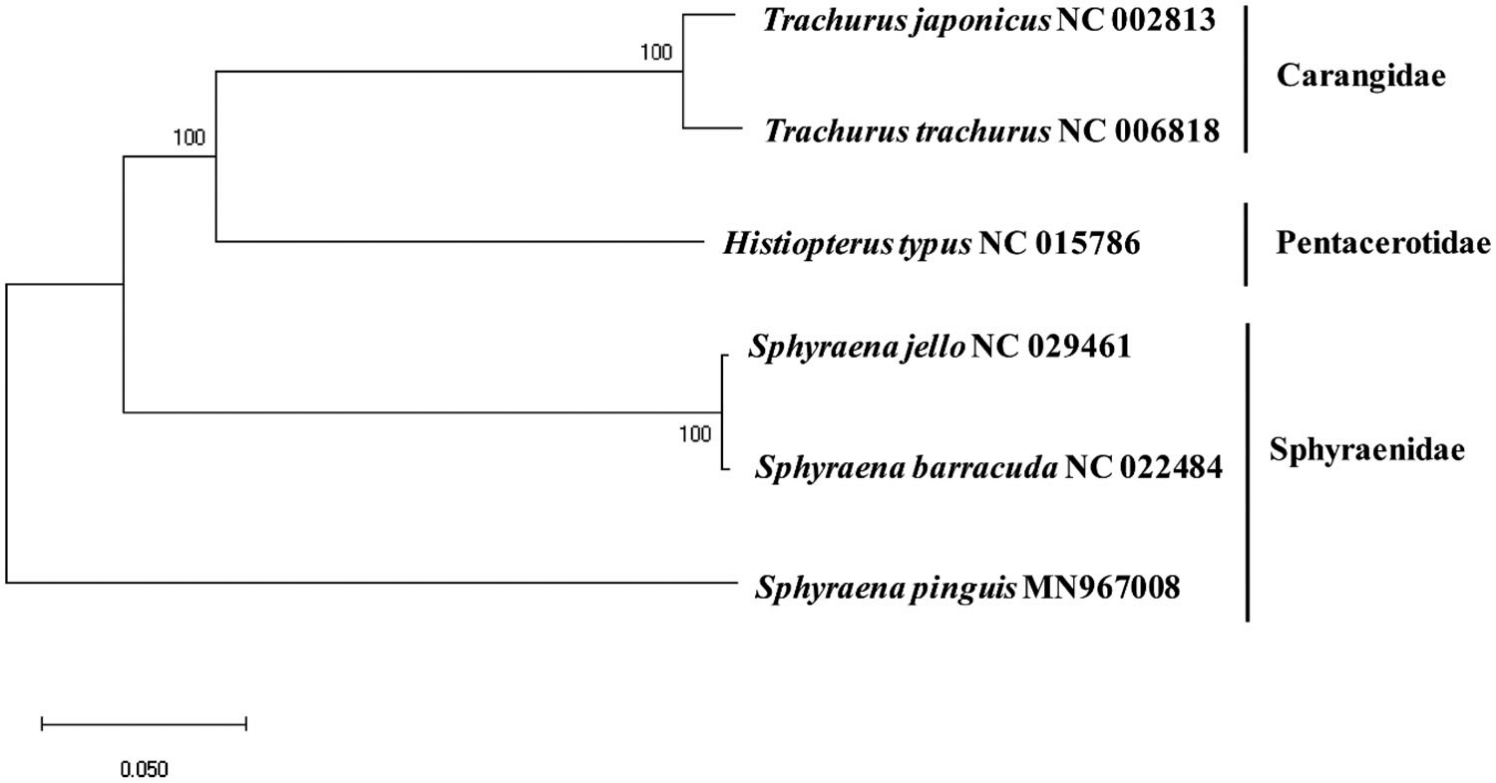
Phylogenetic relationship of *Sphyraena pinguis* among the fish in the order Perciformes: a phylogenetic tree with the complete mitochondrial genome in the order Perciformes displaying with Minimum Evolution (ME) algorithm with 1000 bootstrap replicates. The scientific name of each species along with its GenBank accession number is displayed.

## Data Availability

The data that support the findings of this study are available in [GenBank database] [*Sphyraena pinguis*] at [https://www.ncbi.nlm.nih.gov/nucleotide/], reference number [GenBank Number: MN967008].
